# Promising Natural Polymer-Based Dressings for Diabetic Foot Ulcers: Mechanisms, Preclinical Studies, and Clinical Applications

**DOI:** 10.3390/pharmaceutics18070776

**Published:** 2026-06-25

**Authors:** Yixuan Fang, Jing Wu, Shiyi Sun, Yan Li, Xingwu Ran

**Affiliations:** 1 Department of Endocrinology & Metabolism, West China Hospital, Sichuan University, Chengdu 610041, China; 2 Innovation Research Center for Diabetic Foot, Diabetic Foot Care Center, West China Hospital, Sichuan University, Chengdu 610041, China; 3 Center for High Altitude Medicine, West China Hospital, Sichuan University, Chengdu 610041, China

**Keywords:** diabetes, wound healing, natural polymer, dressing

## Abstract

Diabetic foot ulcers (DFUs) are among the most severe complications affecting diabetic patients, and dressing therapy is one of the standard treatments for DFUs. However, traditional dressings are inadequate for addressing the complex microenvironment of DFUs. Consequently, advanced natural polymer-based dressings have attracted extensive research attention in diabetic foot care due to their biocompatibility, low immunogenicity, and biodegradability. These natural polymer materials include collagen, gelatin, chitosan (CS), hyaluronic acid (HA), alginate, and cellulose. This review systematically analyzes the pathophysiological mechanisms underlying the difficult healing of DFUs and the advantages of natural polymer-based dressings in diabetic wound healing, highlights preclinical studies, and synthesizes evidence from clinical research. Moreover, we pinpoint the challenges associated with these dressings and propose future directions for the improvement of diabetic wound care.

## 1. Introduction

Diabetic foot ulcers (DFUs) represent a critical complication of diabetes, with a global prevalence of 6.3% and a prevalence rate of up to 13% in North America [1]. As defined by the International Working Group on the Diabetic Foot (IWGDF), DFUs are full-thickness skin wounds on the foot that involve at least the epidermis and parts of the dermis, linked to neurological damage and microvascular circulatory disorders caused by hyperglycemia, among other factors [2,3]. Studies indicate that up to 34% of diabetes patients will develop foot ulcers, and these infections often delay the healing process, thereby increasing the complexity of healing [4,5]. Given the chronic and intractable nature of DFUs, the data reveal that the five-year mortality rate of patients with DFUs is approximately 50% [6]. Meanwhile, around 20% of DFU patients undergo amputation, which is associated with a further rise in mortality [7]. Moreover, the medical costs of DFU patients are twice those of diabetic patients without foot ulcers, accompanied by an increase in hospitalization days, home care requirements, and outpatient visits, which place a significant financial burden on individuals, families, and society [8].

Dressing therapy is a widely adopted treatment method throughout the entire management process of DFUs. According to the IWGDF, it is recommended that local care for DFUs include the use of dressings that can manage excessive exudate and maintain a moist wound environment [9]. In comparison with natural polymer-based dressings, traditional dressings have significant drawbacks. For instance, they often fail to maintain an ideal moist environment, and their absorption of wound exudate may result in the dressing adhering to the wound bed, thus causing secondary trauma and other complications during dressing changes [10,11].

Due to the intrinsic limitations of traditional dressings, such as dry gauze, cotton gauze, and bandages [12], advanced natural polymer-based dressings have been widely explored for their potential in promoting diabetic wound healing. These materials include collagen, gelatin, chitosan (CS), hyaluronic acid (HA), alginate, and cellulose. Numerous studies have evaluated the effectiveness of bioactive dressings in wound healing, and each dressing possesses distinct characteristics. However, there are still some limitations in their clinical application. Although several reviews on the application of natural materials in diabetic wounds have been published, they mainly focus on material preparation [12,13]. In contrast, this review focuses on the mechanisms, advantages, and preclinical and clinical applications of natural polymer-based dressings in diabetic wound healing, providing theoretical support for future clinical applications.

## 2. The Pathogenesis of DFUs

The wound healing process typically consists of four overlapping stages [14]. Affected by multiple pathological factors, the diabetic wound healing stages do not progress in an orderly manner, thus hindering the diabetic wound healing process.

### 2.1. Neuropathy and Peripheral Arterial Disease

Research has demonstrated that diabetic patients are confronted with a 50% lifetime probability of developing diabetic neuropathy, which serves as a significant contributing factor to hospitalization and amputation among patients with DFUs [15]. Neuropathy contributes to the development of DFUs through the following mechanisms: (1) neuropathy causes sensory loss, which impairs the perception of trauma and heightens the risk of injury; (2) motor neuropathy gives rise to foot deformities, uneven foot loading, and plantar abrasion, thereby expediting the formation of ulcers; (3) autonomic neuropathy disrupts sweat regulation and skin blood flow, ultimately facilitating the formation of DFUs [16,17]. Additionally, peripheral arterial disease (PAD) is another common factor leading to foot ulcers or amputations [18]. PAD reduces perfusion in the lower limb, leading to chronic limb ischemia, which can result in gangrene and the subsequent progression of DFUs [16]. Meanwhile, PAD is also associated with metabolic imbalances in diabetic patients, such as hyperglycemia and insulin resistance, leading to disruptions in NO homeostasis and other pathological processes [19].

### 2.2. The Impact of Hyperglycemia on Keratinocytes and Fibroblasts

Hyperglycemia can affect the function of keratinocytes in the following manner: (1) hyperglycemia significantly suppresses the activity of keratinocyte, including cell migration and proliferation, thereby delaying the wound re-epithelialization process [20]; (2) the hyperglycemia environment markedly increases the expression of inflammatory molecules in keratinocytes, including IL-1β, TNF-a, and IL-12 [21]; (3) hyperglycemia can disrupt the function of keratinocytes, inhibit the secretion of Human β-defensin-2, and make wounds more susceptible to infection [22]. The transdifferentiation of fibroblasts into myofibroblasts is stimulated by M2 macrophages via TGF-β, and M2 macrophages facilitate fibroblast proliferation and migration [23,24,25]. Nevertheless, a prolonged hyperglycemic environment impedes the conversion of M1 macrophages into M2 cells. This dysregulation not only exacerbates the inflammatory responses at the wound site but also undermines the role of fibroblasts in the wound healing process [26].

### 2.3. The Molecular Mechanism Underlying DFUs

Advanced glycation end-products (AGEs) are molecules produced via the Maillard reaction. Under conditions of hyperglycemia and oxidative stress, AGEs accumulate in the body [27]. The excessive accumulation of AGEs can affect the healing process of DFUs in the following manner: (1) the binding of AGEs to their receptors of AGE (RAGEs) can result in an increased expression of pro-inflammatory genes; (2) AGEs can directly hinder wound repair by reducing fibroblast activity and extracellular matrix (ECM) formation and suppressing granulation tissue formation [28,29].

Oxidative stress refers to an imbalance between pro-oxidant and antioxidant systems, with reactive oxygen species (ROS) serving as an indicator of oxidative stress [30]. Under physiological conditions, ROS participate in wound healing by regulating vasoconstriction and facilitating the recruitment of inflammatory cells to the wound site [31]. Nevertheless, an excessive amount of ROS results in an aggravated inflammatory state [32]. It is important to note that the ongoing hyperglycemic environment further promotes the increased buildup of ROS [33]. This accumulation initiates continuous oxidative stress and lipid peroxidation, leading to cellular damage and aging, which hinders the wound healing process [34].

Macrophages, neutrophils, fibroblasts, and other cell types synthesize matrix metalloproteinases (MMPs), which play a central role in ECM remodeling [35]. However, in diabetic patients, metabolic disorders in the microenvironment disrupt the balance between MMPs and tissue inhibitors of metalloproteinases (TIMPs), thus affecting the remodeling of the wound ECM [36]. Moreover, numerous studies have shown that the upregulation of MMP-9 contributes to the impairment of wound healing, making it a potential target for the treatment of DFUs [35,37,38,39]. Therefore, the targeted regulation of MMP levels is beneficial for wound healing.

**Figure 1:**
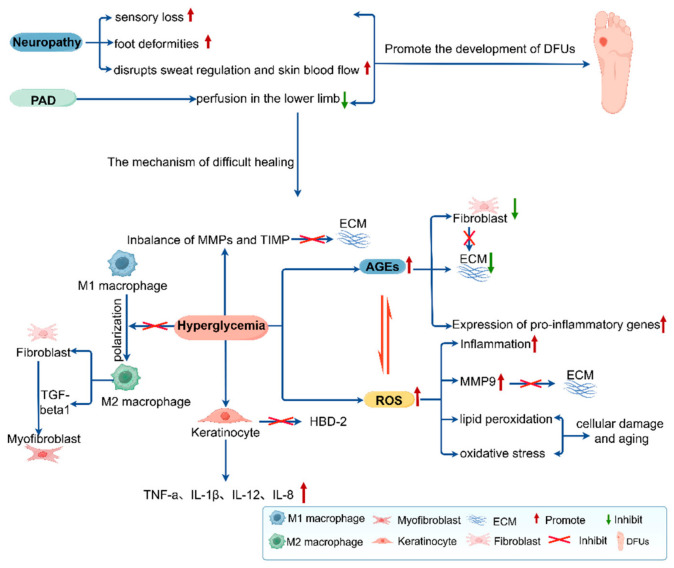
Mechanisms of risk factors associated with impaired healing in DFUs (generated using Figdraw). PAD: peripheral arterial disease; DFUs: diabetic foot ulcers; ECM: extracellular matrix; AGEs: advanced glycation end-products; ROS: reactive oxygen species; HBD-2: Human β-defensin-2; MMPs: matrix metalloproteinases; TIMPs: tissue inhibitors of metalloproteinases.

Under in vivo hyperglycemic conditions, AGEs, oxidative stress, and MMPs interact with each other. AGEs can bind to RAGEs, which are expressed by endothelial cells, leading to an increase in the formation of ROS. Moreover, the hyperglycemic environment elevates the levels of ROS in the body, thereby further promoting the intracellular formation of AGEs [30,40]. Simultaneously, persistent hyperglycemia induces oxidative stress, which further enhances the activity of MMP-9 [41]. We summarized the mechanisms of risk factors associated with impaired healing of DFUs in Figure 1.

## 3. Characteristics and Preclinical Studies of Natural Polymer-Based Dressings

The aforementioned pathological mechanisms indicate that the healing of DFUs requires the consideration of complex mechanisms, including hyperglycemia, oxidative stress, AGEs, and other factors. Dressings based on natural polymers have become a significant area of research because of their remarkable properties, which provide numerous advantages consistent with the complex healing mechanisms of DFUs. The following natural polymers are presented: collagen, gelatin, CS, HA, alginate, and cellulose.

### 3.1. Collagen and Gelatin

Collagen, a highly prevalent structural protein distributed throughout the human body, functions as a fundamental component of the ECM of the skin [42]. Collagen consists of three polypeptide chains that form a right-handed triple helix with Gly–Pro–Hyp repeats. These chains have the ability to assemble into fibrils and larger fibers, thus endowing mechanical strength [43]. Collagen can be predominantly classified into natural collagen and artificially synthesized recombinant collagen. The common sources of natural collagen encompass animals such as pigs and cattle, as well as marine organisms. In contrast, recombinant collagen is artificially synthesized through recombinant technology [43,44]. Recombinant collagen can be obtained from prokaryotes, mammals, or insects [45]. Collagen derived from animals may present risks such as disease transmission, allergic reactions, microbial contamination, or religious restrictions. Conversely, recombinant collagen mitigates these associated risks [45]. Nevertheless, a study has indicated that one of the factors constraining the large-scale production of recombinant collagen is the requirement for the hydroxylation of proline after translation [46]. To address this issue, research has indicated that proline hydroxylation can be accomplished in recombinant human collagen by coexpressing recombinant collagen and human prolyl 4-hydroxylase [47]. At the same time, regulatory authorities have issued guidelines on the standards for recombinant collagen, including raw materials, physical and chemical properties, technical requirements, packaging, transportation, and storage, to ensure the stability of the product [48]. Generally, its mechanical characteristics can be modified via polymer synthesis to adapt to the application form. For instance, research has found that the incorporation of β-glucan into collagen can significantly improve its mechanical tensile strength when it is used as a wound dressing [49].

Collagen participates in the entire wound healing cascade as follows: (1) in the hemostasis phase, collagen participates in platelet adhesion [50]; (2) during the inflammatory phase, it contributes to suppressing the expression of inflammatory mediators, attracts neutrophils through chemotaxis, and simultaneously alleviates oxidative stress [51,52]; (3) in the proliferation and re-epithelialization phase, collagen recruits fibroblasts, which produce more collagen to build the ECM [53,54]. Meanwhile, the progressive deposition of type I collagen, which substitutes the initial type III collagen network, results in scar formation during wound healing.

Collagen dressing possesses the ability to enhance wound healing. For example, research has indicated that two bovine-derived collagen wound dressings accelerated the healing process by acting as competitive substrates that bind to MMP-2 and MMP-9, thereby facilitating wound repair [55]. Meanwhile, advanced collagen-based dressing can improve the wound healing mechanism to promote wound healing in preclinical studies. For instance, Yang et al. constructed a smart responsive hydrogel consisting of recombinant human type III collagen and extracellular vesicles, which promoted fibroblast proliferation and migration and relieved oxidative stress by reducing free radicals. Additionally, it promoted angiogenesis and regulated inflammation through the NF-κB and YAP pathways [56].

Gelatin is derived from the denaturation of collagen, which is primarily sourced from animals. It adopts a random coil structure [57]. When compared with collagen, it demonstrates higher solubility and lower antigenicity. Cell adhesion is facilitated by the sequence of arginine–glycine–aspartic acid (RGD), and MMP target sequences that are conducive to cell remodeling [58]. Gelatin methacrylate (GelMA), a derivative of gelatin, not only retains the inherent advantages of gelatin, such as facilitating cell adhesion and possessing good biocompatibility, but also exhibits photopolymerization properties [58].

Due to its excellent biocompatibility and degradability, gelatin is often crosslinked with other polymers to load drugs or molecules, thereby fulfilling multiple functions. For instance, Yang et al. developed a gelatin-based injectable nanocomposite hydrogel that incorporated a metal–organic framework. The dressing exhibited biocompatibility, mechanical properties, mechanical stability, flexibility, favorable expansibility, optimized pore characteristics, degradability, and the capacity to achieve slow release of drugs. The injectable nanocomposite hydrogel could scavenge ROS, decrease the levels of IL-6 and IL-10, and enhance the expression of CD31 and α-SMA in the diabetic model [59].

Overall, the distinct advantages of collagen and gelatin highlight their potential for a broad spectrum of medical applications. For example, the type I collagen-based product CellerateRx represents a therapeutic option specifically approved by the US Food and Drug Administration (FDA) for the management of DFUs. A study has shown its capacity to promote the healing of DFUs [60]. Researchers often crosslink them with other polymers to expand their applications, such as in the development of various types of dressings for diabetic wounds, including hydrogels and cell scaffolds. For example, a gelatin-based injectable hydrogel can accommodate the irregular wounds of diabetic ulcers [59].

### 3.2. Chitosan

CS, a polysaccharide derived from the partial deacetylation of chitin, naturally exists in crustaceans [61]. The antibacterial efficacy of CS is contingent upon specific parameters, particularly the degree of deacetylation, molecular weight, and PH. An escalating degree of deacetylation enhances the number of amino groups, which enhances antibacterial activity [62]. This antibacterial activity is mediated through several mechanisms: (1) cationic amino groups electrostatically bind to the negatively charged bacterial cell membrane; (2) CS binds to microbial DNA, thus inhibiting protein synthesis and inducing microbial death; (3) CS chelates metal ions, which disrupts normal microbial metabolism [63]. CS exhibits broad-spectrum antibacterial properties, which further enhances its application in the treatment of DFUs [64]. Secondly, CS, by virtue of its cationic amino groups formed through deacetylation, exhibits remarkable hemostatic properties, such as promoting the aggregation of erythrocytes and platelets. Simultaneously, it facilitates vasoconstriction through the absorption of nitric oxide mediated by erythrocyte aggregation [65]. Additionally, CS exhibits mild antioxidant activity because its hydroxyl and amino groups, which neutralize free radicals, are partially constrained by hydrogen bonding in the polymer structure [66]. Finally, CS is soluble in acidic solutions because its amino groups are protonated, whereas, in neutral water, uncharged chains tend to aggregate, resulting in reduced solubility [67]. This solubility limitation can be alleviated via the chemical modification of CS to introduce carboxymethyl functional groups, thus forming carboxymethyl chitosan (CMCS), which significantly expands its application potential [68].

Due to its excellent physiological properties, CS has attracted considerable research attention within the realm of wound healing. Meanwhile, the FDA has approved CS as a safe compound [69]. Furthermore, there is already a wide variety of CS products available in the market, such as Axiostat^®^, Celox™, ChitoClear^®^, HemConTM, plus^®^, and Chitoderm, among others [70,71], which further demonstrates the availability of CS-based dressings.

As previously mentioned, CS demonstrates excellent antimicrobial properties. Moreover, CS can be chemically modified to incorporate various functional groups, thereby enhancing its antibacterial properties. These modifications not only retain the unique features of CS but also impart appropriate porosity and mechanical strength to the dressings [72,73]. For instance, Liu et al. fabricated dual layer microneedle patches by blending CMCS with other materials, which exhibited excellent antibacterial efficacy in diabetic wounds [74]. Additionally, CS-based dressings can achieve superior antibacterial effects by incorporating antibacterial agents or employing photothermal therapy. For instance, in vitro experiments exhibited that the quaternized CS composite hydrogel demonstrated antibacterial efficiencies of over 60% against Staphylococcus aureus and Escherichia coli, which further increased to over 99% when exposed to infrared irradiation [75].

Simultaneously, CS-based dressings can serve as carriers for stem cells or drugs to achieve localized treatment of diabetic wounds in diabetic animal models through mechanisms of anti-inflammation, antioxidation, and angiogenesis. Researchers constructed a hydrogel network by using CMCS loaded with deferoxamine (DFO) and demonstrated a decrease in the levels of pro-inflammatory factors, and an increase in the levels of IL-4, IL-10, and vascular endothelial growth factor (VEGF) in diabetic mouse wounds [76]. Similarly, Xing et al. fabricated a CS-based hydrogel loaded with stem cells and observed similar mechanisms in a diabetic wound [77].

Many preclinical studies have established that dressings based on CS, including those incorporating therapeutic agents, promote the repair of diabetic wounds. Preclinical studies indicate that their efficacy results from enhanced wound healing through multiple mechanistic pathways. By integrating the properties of CS with the ability to carry molecules or drugs, these dressings effectively tackle the problem of wound healing, including reducing inflammation, promoting angiogenesis, and providing antibacterial action. Therefore, CS-based dressings represent a potential therapeutic approach for diabetic wound healing.

### 3.3. Hyaluronic Acid

HA, a fundamental constituent of the ECM, is a natural polymer that belongs to the polysaccharide class of glycosaminoglycans [78]. HA is a linear polysaccharide composed of repeating disaccharide units. It is characterized by an abundance of hydroxyl and carboxyl groups and has the capacity to form diverse secondary and tertiary structures [79]. Therefore, it possesses unique attributes such as viscoelasticity and rheological properties [80]. HA is sourced either via animal tissue extraction or microbial fermentation, with microbial fermentation currently being the primary source. HA exhibits properties that are contingent upon its molecular weight: (1) Low-molecular-weight HA is considered to have the ability to regulate cancer-associated cells. It can more easily penetrate the stratum corneum and reach the dermis, and it can be applied in cosmetics. (2) High-molecular-weight HA has been reported to possess anti-inflammatory properties and a high viscoelasticity. Nevertheless, it is readily trapped in the stratum corneum, and it can be used in ophthalmic surgery and joint syringes [81,82,83,84]. The biological activities of HA are mediated through its interactions with multiple molecules, including CD44 and the receptor for hyaluronate-mediated motility (RHAMM). CD44 facilitates the migration of fibroblasts to the wound site, and the activation of RHAMM stimulates the proliferation of fibroblasts. Meanwhile, HA influences angiogenesis through both CD44 and RHAMM [85,86].

Researchers often enhance the function of HA-based hydrogel by incorporating it with other polymers. For example, Yuan et al. designed a hydrogel scaffold via the covalent crosslinking of polyurethane and HA. The hydrogel scaffold exhibited high structural stability, improved elasticity, and controllable degradation characteristics [87]. HA undergoes chemical modification, crosslinking, and binding with a variety of therapeutic molecules. Its hydrophilic nature and gel forming ability ensure excellent drug loading efficiency and controlled release. Furthermore, HA demonstrates viscoelastic properties and hygroscopicity, which contribute to the regulation of tissue moisture and osmotic balance, thereby enhancing the functionality of cells and collagen fibers in a stable and moisturized environment [88]. These properties make HA an ideal scaffold for multifunctional composite dressings. Additionally, HA is considered safer than many other natural polymers due to its low immunogenicity, and several HA-based products have received FDA approval for commercialization [89].

Currently, a large number of HA-based dressings have been designed to explore their therapeutic efficacy on diabetic wounds. HA-based dressings can promote wound healing through multiple mechanisms in diabetic animal models. For example, an injectable HA-based hydrogel with glucose-responsive metformin upregulates anti-inflammatory factors, downregulates pro-inflammatory factors, promotes M2 macrophage polarization, and enhances the expression of CD31 and VEGF in diabetic rats [90]. Meanwhile, HA can form a stable drug or molecular carrier. For example, Hua et al. developed a composite hydrogel based on hyaluronic acid methacryloyl (HAMA) and a functionalized polymer, successfully achieving the continuous release of exosomes, and enhancing their in vitro activity. The hydrogel releases exosomes in a sustainable manner to facilitate wound healing in diabetic mice through anti-inflammatory activity, the promotion of angiogenesis, and M2 macrophage polarization [91].

Although numerous preclinical studies have shown that HA-based dressings effectively promote diabetic wound healing, their clinical translation is limited. Therefore, further large-scale clinical studies are needed to translate the clinical application of HA-based dressings.

### 3.4. Alginate

Alginate is a natural polymer that belongs to the polysaccharide family. It is derived from algae (predominantly brown algae) and a variety of bacteria [92]. The molecular architecture of alginate serves as the primary determinant of its physical properties. Modifications in the content of GG, MM, and M/G will lead to variations in the viscosity and elasticity of alginate. For example, different elastic hydrogels are formed depending on the GG and MM content [93]. Meanwhile, the M/G ratio and its structural configuration govern the stability and phenotype, while also modulating the hydrogel function of the carrying and delivering [94]. The FDA has approved the utilization of alginate in medicine, food, and other fields. Alginate not only demonstrates low immunogenicity and biocompatibility, but also undergoes biodegradation into non-toxic substances [95]. Moreover, alginate is abundant in calcium and zinc ions, which can facilitate blood clotting [96]. Simultaneously, alginate is regarded as having anti-inflammatory, antimicrobial, and antioxidant properties, making it a suitable natural polymer for diabetic wound dressings [97,98,99].

Natural alginate can be modified to obtain more features. Research has indicated that angiogenesis can be promoted by modifying its functional groups. For instance, alginate was modified with sulfate groups to promote binding between VEGF and VEGF receptors [100]. Additionally, alginate is capable of regulating drug release. The degradation rate and drug release rate of alginate under different PH conditions can be regulated through the adjustment of chemical modification ratios [101].

A single alginate dressing promotes wound healing in diabetes. For example, Lu et al. found that alginate derived from Sargassum can significantly accelerate the healing of diabetic wounds. The study has confirmed that the mechanism may involve the activation of Nrf2 to enhance antioxidant capacity, the regulation of VEGF to promote angiogenesis, and a substantial reduction in skin inflammation in both in vivo and in vitro experiments. This further indicates that alginate dressings are highly appropriate for diabetic wounds [102]. Moreover, alginate can be fabricated into hydrogels, skin scaffolds, patches, and other forms to encapsulate drugs, molecules, and so on, which provide a more efficient mode of administration for facilitating wound healing. Research has shown that the utilization of polyethylene glycol sodium alginate nanogels to encapsulate platelet lysate can enhance the therapeutic efficacy on diabetic wounds compared to the sole use of platelet lysate, while demonstrating excellent sustained molecule release [103].

Alginate-based dressings are capable of loading novel therapeutic agents, thereby forming wound dressings that can promote diabetic wound healing by regulating inflammation levels, ROS levels and so on. For example, a sodium alginate (SA)-based hydrogel designed for the sustained release of Netrin-1 decreased ROS levels and promoted the proliferation of fibroblasts [104]. Additionally, an SA-based hydrogel encapsulating mesenchymal stem cells can upregulate BMAL1, thereby inhibiting neutrophil extracellular trap (NET) formation and enhancing wound healing [105]. These dressings partially address wound healing impairments in diabetic wounds by alleviating excessive oxidative stress, shortening the prolonged inflammatory phase, and supporting tissue regeneration.

However, the alginate-based dressings formed through different synthesis methods or loaded with different bioactive substances exhibit distinct characteristics. For example, a hydrogel was fabricated through the crosslinking of nanoparticles with SA, which demonstrated antibacterial activity, electrical conductivity, and responsiveness to infrared light [106]. In contrast, the hydrogel synthesized by Lin et al. featured a porous structure and exhibited controlled drug release [105].

Overall, alginate-based dressings exhibit substantial potential in the treatment of diabetic wounds, particularly as drug delivery systems. Preclinical studies indicate that they can facilitate the progress of diabetic wound healing.

### 3.5. Cellulose

Cellulose ranks among the most abundant and widely distributed polysaccharides in nature [107]. It is a high-molecular-weight polysaccharide composed of β-1,4-linked glucose units. Characterized by a high hydroxyl content, a crystalline architecture, and a supramolecular organization, cellulose exhibits water insolubility, hydrophilicity, and chemical modifiability [108]. Natural cellulose can be obtained from plants, bacteria, and minerals. The majority of commercial cellulose is mainly derived from wood, leaves, cotton, and so on [109,110]. Similar to other natural polymers, cellulose possesses biological properties, such as biodegradability, biocompatibility, and low toxicity. Consequently, it has become one of the most intensively researched materials in the field of natural dressings for wound healing. To broaden the application scope of cellulose, chemical, physical, or biological functionalization can be conducted, thereby expanding the application range of cellulose [111]. Carboxymethyl cellulose (CMC) is obtained via the chemical etherification of native cellulose. It maintains the inherent biological properties of cellulose and promotes the proliferation and migration of fibroblasts [112]. Due to its characteristics, CMC is commonly utilized as a drug or molecule carrier. For instance, Ren et al. applied CMC to load metal nanoparticle ions, fabricating microneedle dressings that notably inhibited bacterial growth, thus facilitating the healing of infected wounds in diabetic mice [113].

Bacterial cellulose (BC) is a highly pure form of cellulose biosynthesized by bacteria [114]. Conversely, plant cellulose (PC), which is isolated from plants, contains impurities due to the presence of other polymers [115]. The microfibrils that constitute BC are approximately 100 times smaller than those of PC, with a width of less than 100 nanometers [107]. Although BC and PC have similar chemical structures, BC exhibits higher level of cellulose purity [107,116]. Moreover, BC demonstrates remarkable water-retention properties, which contribute to maintaining a moist environment, absorbing exudates, and effectively facilitating re-epithelialization and tissue regeneration [117]. When compared with PC, BC demonstrates superior structural, functional, and mechanical properties [114]. BC possesses a three-dimensional network structure that can promote cell proliferation, penetration, and growth of connective tissue cells [118]. Additionally, it can prevent wound infection, ensure wound permeability, and maintain a high water content, which renders it suitable for the management of diabetic wounds [117]. Medical sterile gauze, a traditional wound dressing commonly employed for wound care, is generally derived from PC. Hsu et al. employed BC and gauze for wound treatment in diabetic mice. The results indicated that BC had superior therapeutic efficacy, outperforming gauze in terms of healing speed, tissue proliferation, and antimicrobial activity [119]. Therefore, BC is a promising wound dressing for diabetes.

Composite cellulose dressings, which incorporate a variety of bioactive components, can facilitate diabetic wound healing by promoting angiogenesis, anti-inflammatory activity, antioxidant activity, and so on. For example, BC-based hydrogels loaded with copper nanoparticles (CuNPs) enhanced angiogenesis and collagen deposition in the wounds of diabetic rats under electrical stimulation [120]. Similarly, a gel composed of cysteamine-modified cellulose nanospheres enhanced wound repair in diabetic mice by decreasing the levels of IL-6, IL-1β, and TNF-α, while augmenting the expression of VEGF [121]. The functional properties of these cellulose-based dressings depend on the loading agents: dressings loaded with CuNPs exhibit antibacterial activity, while dressings containing MXene provide antioxidant and conductive capabilities [120,122].

BC is generally cultivated in the laboratory via static cultivation methods, which are both costly and time-consuming. Research has indicated that the BC yield was 7.81 g/L after 9 days of static culture on the medium [123]. Consequently, in industrial large-scale production processes, bioreactors are generally employed to reduce costs and enhance yields. Nevertheless, it remains challenging to control the properties of BC on an industrial scale with existing technology [124]. Overall, BC still requires further exploration to enhance production efficiency and properties in large-scale production for the purpose of achieving large-scale clinical applications.

### 3.6. Characteristics of Natural Polymers

**Figure 2:**
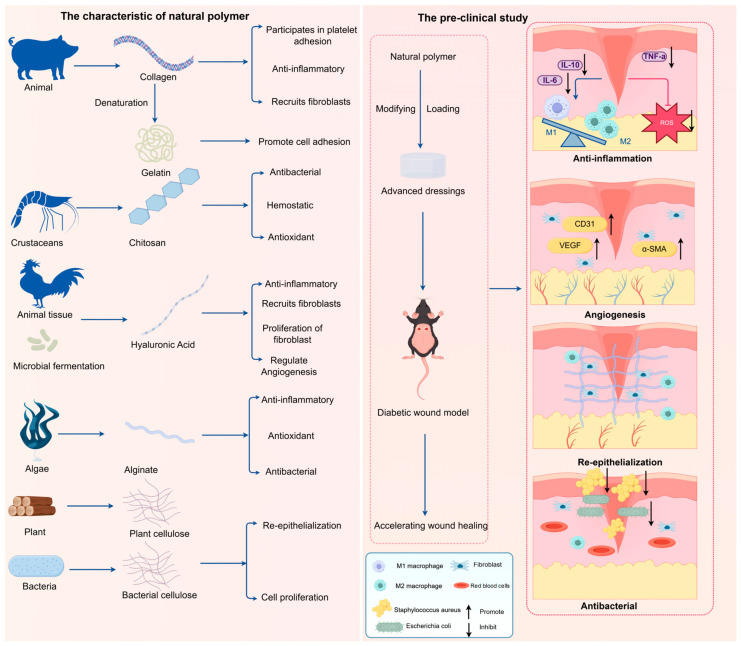
Characteristics and preclinical studies of natural polymer-based dressings (generated using Figdraw).

As previously stated, the optimal dressing for chronic diabetic wounds should fulfill the following criteria [125]: (1) maintain a moist environment while adsorbing exudate; (2) exhibit excellent antibacterial properties to reduce the occurrence of infection; (3) demonstrate high-level biocompatibility; (4) be capable of transporting and storing medications; (5) be biodegradable or easily replaceable; (6) have low immunogenicity; and (7) be suitable for large-scale production and involve low economic costs. To illustrate more clearly the characteristics of each natural polymer, we summarize their structure, features, and applications in Table 1. Meanwhile, we comprehensively present the sources of natural polymers, the mechanisms in wound healing and the mechanisms of preclinical research in Figure 2.

The results presented in Table 1 and Figure 2 indicate that each natural polymer has similar characteristics and unique advantages. Firstly, natural polymers demonstrate low immunogenicity and excellent biocompatibility. Secondly, due to the variability of their molecular structures, each natural polymer has different biological characteristics. For example, both HA and alginate show high hydrophilicity because they both have a substantial quantity of hydrophilic groups. Meanwhile, the presence of cationic amino groups in CS is one of the reasons that it has antibacterial and hemostatic properties. Additionally, Figure 2 depicts the mechanism by which different natural polymers are involved in wound healing. For example, collagen and HA are essential components of the ECM in skin tissue. Therefore, they possess the characteristic of promoting re-epithelialization. Preclinical studies have shown that advanced natural polymer-based dressings can promote wound healing through various mechanisms, including anti-inflammatory activity, antibacterial activity, re-epithelialization and angiogenesis promotion. Different natural polymer-based dressings can exhibit the same characteristics through different modifications or by carrying similar bioactive molecules. For example, BC is a non-antibacterial natural polymer. However, when BC is combined with CuNPs, the dressing can achieve antibacterial effects [126]. Therefore, natural polymers have different characteristics, but, as shown in Figure 2, researchers can modify the polymers according to their needs to develop multifunctional dressings that can promote wound healing through various mechanisms.

**Table 1:** Characteristics of natural polymers.

Natural Polymer	Structural Characteristics	Biological Properties	Application	References
Collagen	Polypeptide chains containing Gly–Pro–Hyp form a triple helix capable of self-assembling into fibrils and macroscopic fibers	Low immunogenicity, high biodegradability, high water absorption capacity	Wound dressings, artificial dermis skin replacement, never regeneration, drug delivery, tissue regeneration	[43,57]
Gelatin	Randomly coiled polypeptide chains	Biocompatibility, biodegradability, low immunogenicity	Wound dressing, artificial skin, drug carrier	[57,127]
Chitosan	Cationic amino groups containing a large number of active functional groups	Biocompatibility, antibacterial properties, hemostatic capabilities, promotes cell adhesion, antioxidant, anti-inflammatory, antitumor	Wound dressing, tissue engineering, bio-adhesives	[128,129]
Hyaluronic acid	Repeated disaccharide units; a substantial quantity of hydrophilic groups	High water absorption, high water solubility, low immunogenicity, improves the viability of fibroblasts and keratinocytes, facilitates angiogenesis	Drug delivery, cell adhesion, joint lubrication, tissue engineering, wound dressing	[85,89,130]
Alginate	β-D-mannuronic acid and α-L-guluronic acid are linked either homogenously or heterogeneously through 1–4 glycosidic bonds; a substantial quantity of hydrophilic groups	Biocompatibility, biodegradability, low immunogenicity, water absorption capacity, high water solubility, hemostasis, antioxidant, angiogenesis, antibacterial, anti-inflammatory	Drug delivery, tissue engineering, wound dressing, immobilized cells	[131]
Bacterial cellulose	A fibrous structure composed of repeated glucose units; three-dimensional nanofiber network; a substantial quantity of hydroxyl groups	Biocompatibility, non-toxicity, low immunogenicity, high hydrophilicity, provides mechanical protection for wounds	Artificial skin, blood vessels, wound dressing, drug delivery	[132,133]
Plant cellulose	Composed of extended β-D-glucose chains with aligned chains forming microfibrils stabilized by hydrogen bonds	Biocompatibility, non-toxicity, low immunogenicity, moderate hydrophilicity, a low cost and abundant source	Drug delivery, wound dressings, tissue engineering	[108,134]

**Figure 3:**
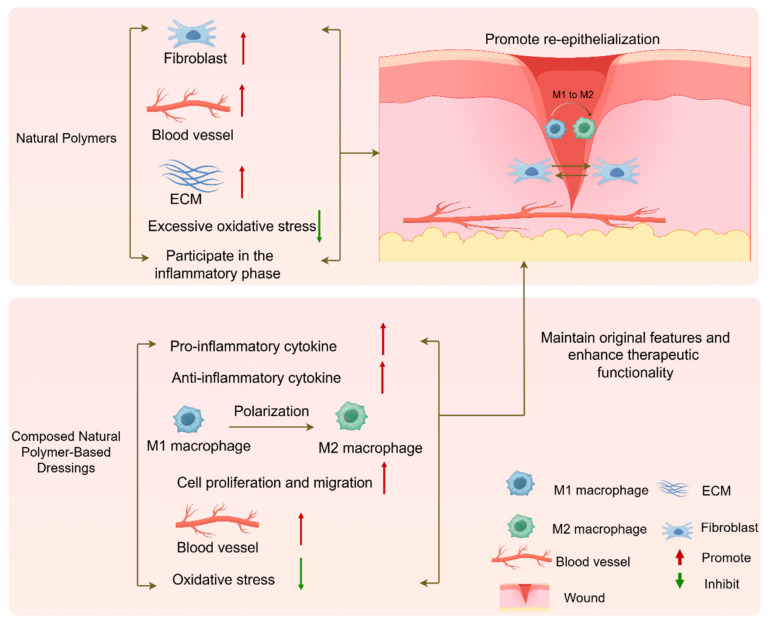
Natural polymers and dressings based on natural polymers for the regulation of healing mechanisms (generated using Figdraw). ECM: extracellular matrix.

In general, DFUs pose challenges in terms of healing because of their complex micro-environment, which primarily encompasses hyperglycemia, persistent inflammatory responses, infections, and oxidative stress, among other factors. Natural polymers and their composite dressings can promote the healing of diabetic wounds through diverse mechanisms. The mechanisms by which natural polymers and their composite dressings promote wound healing are summarized in Figure 3.

## 4. The Advantages of Dressings Based on Natural Polymers in the Treatment of DFUs

The utilization of drugs, bioactive molecules, exosomes, or stem cell therapies for the treatment of diabetic wounds has emerged as a research focus. However, the application of novel therapeutic agents to localized wound areas remains a lot of challenge. This includes the use of antimicrobial drugs/molecules for infection management, various growth factors for tissue restoration, and genes for targeted therapy. Natural polymers enable the controlled release of drugs/small molecules at localized wound sites. Meanwhile, natural polymers can form structures that closely resemble natural skin, thus further enhancing the repair of DFUs. In this context, the advantages of natural polymer-based dressings in the treatment of diabetic wounds will be discussed from these two perspectives.

### 4.1. Multifunctional Carriers Designed for Drugs or Molecular Substances

Firstly, natural polymers are outstanding carriers that can effectively preserve the activity of encapsulated substances. For example, Kong et al. significantly enhanced the in vitro activity of stem cells by using photo-crosslinked recombinant human type III collagen to form a hydrogel encapsulating stem cells [135]. Furthermore, the biodegradability of natural polymer dressings ensures the biocompatibility and low immunogenicity of the drug carrier. At the same time, it prevents the side effects caused by frequent dressing changes. Secondly, natural polymers enable the controlled delivery of encapsulated drugs or functional molecules: (1) They improve the bioavailability of therapeutic agents. For example, CS can increase the retention time of drugs at the site of administration [136]. (2) Natural polymers can gradually release the carried molecules, thus avoiding excessively high local concentrations of drugs or molecules. For example, exosomes derived from mesenchymal stem cells are regarded as an effective biological therapy for DFUs [137]. Nevertheless, direct application to the wound site may not guarantee sustained exosome release. Yu et al. developed an injectable HA-based hydrogel loaded with mesenchymal stem cell-derived exosomes, which maintained a stable exosome release over a 14-day period [138].

The implementation of multifunctional dressings can be achieved through the modification of natural polymers. More than half of patients with DFUs suffer from diabetic foot infections, which leads to an increased hospitalization and amputation rate among DFU patients [139]. Therefore, infection control is a critical step in treatment. Natural polymers can be engineered to improve antimicrobial efficacy. For instance, although CS inherently exhibits antimicrobial activity, Wang et al. further enhanced the antimicrobial performance of CS hydrogels by grafting quaternary ammonium and catechol moieties onto the CS backbone [140]. Additionally, the complex microenvironment of chronic diabetic wounds presents a challenge during the treatment process. However, current research has shown that, by modifying natural polymers to achieve a pH- and ROS-responsive release of encapsulated drugs/molecules, dressings can release drugs according to the specific microenvironment of DFU patients, thereby enabling personalized treatment [141,142,143]. For example, a pH/glucose dual-responsive hydrogel can be synthesized using sodium alginate, poly (vinyl alcohol), and tannic acid. This hydrogel releases antimicrobial agents within the low-pH and high-glucose microenvironment of infected diabetic wounds, and in vivo studies have demonstrated its effective eradication of Staphylococcus aureus [144]. Not only can it regulate the release of drugs, but it can also control the release of extracellular vesicles, allowing for the local on-demand delivery of extracellular vesicles. Researchers constructed a hydrogel by using aminophenylboronic acid and recombinant collagen to encapsulate plant-derived extracellular vesicles. The results demonstrated that smart responsive hydrogel accelerates vesicle release under conditions of acidic pH, elevated ROS levels, and high glucose concentrations, thereby promoting diabetic wound healing [56].

### 4.2. Similarity Between the Extracellular Matrix and Regenerative Potential

Due to their biocompatibility, natural polymers do not induce inflammation or severe inflammatory responses, as opposed to the majority of synthetic materials. Their biodegradability and non-toxicity further promote tissue formation, resulting in their widespread application in the field of tissue engineering [145]. Chen et al. constructed a decellularized dermal matrix HA sponge scaffold by mixing a porcine acellular dermal matrix with HA. This 3D scaffold exhibited excellent stability, hydrophilicity, and mechanical properties [146]. Natural polymers are able to mimic the ECM and form biomimetic skin scaffolds. These scaffolds can transport bioactive substances, maintain a moist wound environment, and carry bioactive substances. Therefore, they enable precise control of the wound healing process and further enhance tissue function. For example, researchers fabricated a biomimetic skin collagen scaffold using type I collagen, simultaneously combining genes for basic fibroblast growth factor (bFGF) derived from human umbilical cord mesenchymal stem cells (hUCMSCs). This scaffold exhibited distinct advantages. Firstly, it notably promoted the activity and proliferation rate of the loaded MSCs. Secondly, the biomimetic skin scaffold synergistically enhanced the wound healing effect of hUCMS-bFGF genes on diabetic skin wounds [147]. Meanwhile, the utilization of natural polymers to construct a microenvironment similar to natural tissues, thereby forming a double-layer scaffold structure, not only mimics real skin conditions and maintains a moist environment for wounds, but also facilitates cell passage through pores, cell proliferation, and wound healing [148]. For instance, Shen et al. constructed a bilayer biomimetic skin scaffold by combining BC and gelatin, which has two functions: (1) preventing microbial penetration and (2) effectively carrying bioactive molecules. Moreover, in vitro experiments have demonstrated that it can effectively support cell growth, adhesion, proliferation, and regeneration [149].

## 5. Clinical Applications and Clinical Scenarios of Dressings Based on Natural Polymers

### 5.1. Registered Clinical Trials of Dressings Formulated with Natural Polymers

To conduct an in-depth exploration of the clinical research on natural polymer-based dressings for DFUs, a search of the clinicaltrials.gov database (https://clinicaltrials.gov/, accessed 16 January 2026) was conducted from inception to January 2026. Subsequently, the clinical trials were summarized (Table 2). Nevertheless, no registered clinical studies concerning gelatin-based dressings and BC-based dressings for diabetic wounds were identified. These trials are categorized into four status groups: completed, recruiting, terminated, and unknown.

Initially, 19 studies on the utilization of collagen dressings for DFU were identified. Among these studies, 11 completed clinical trials primarily explored the therapeutic effects of collagen-loaded drugs and target genes on DFUs. For instance, the Tissue Repair Company conducted a study utilizing type I collagen gel loaded with platelet derived growth factor B to evaluate its efficacy in the healing of diabetic skin wounds. A total of 21 DFU patients were enrolled in the study (NCT00065663). In another study, the University Hospital of Geneva assessed gentamicin-loaded collagen sponges in 88 patients with infected DFUs (NCT01951768), with patients receiving systemic antibiotics serving as the control group. However, no significant improvement was observed with the use of gentamicin-loaded collagen sponges [150]. Bioinspired dressings, which mimic the structure and function of natural tissues to exhibit unique biological activity and excellent biocompatibility, have become a research focus in wound care [151]. Two clinical trials on clinicaltrials.gov are related to collagen-based bioinspired dressings. The objective of one trial is to compare high-purity type I collagen-based skin substitutes (HPTC) with dehydrated human amniotic/chorion membrane in DFU patients (NCT07046403), with the aim of providing evidence for subsequent DFU treatment. Meanwhile, another study is recruiting patients with DFUs to evaluate the treatment efficacy of collagen extracellular matrix scaffolds loaded with antimicrobial agents (NCT06618612).

Secondly, four studies related to CS were retrieved. Among these studies, one has concluded clinical research, two have discontinued clinical research, and the status of one remains unknown. The completed clinical trial, designed by the University of Guadalajara, was a study encompassing 68 patients with DFUs. Participants were administered either CS hydrogel in combination with isosorbide dinitrate, isosorbide dinitrate spray, or a placebo to assess the therapeutic potential of CS–isosorbide dinitrate for the treatment of DFUs (NCT02789033).

Ultimately, we independently conducted a summary of studies on HA-based, alginate-based, and cellulose-based dressings in the context of DFUs.

### 5.2. Selection of Dressings

The selection of dressings in clinical practice depends on the type of wound, as well as the patient’s individual differences, wound condition, and economic factors, all of which are crucial considerations. Debridement is considered a key treatment for DFUs, as it effectively removes necrotic, devitalized, or severely infected tissue, creating a clean wound bed [152]. Once the wound bed is properly prepared, the selection of an appropriate dressing, whether it is a conventional dressing or a novel natural polymer-based dressing, is essential for facilitating the healing process.

When choosing dressings, natural polymer materials can provide both traditional and innovative options. Therefore, Table 3 below delineates the advantages and disadvantages of conventional dressings in contrast to emerging natural polymer-based dressings and provides guidance for clinical practice.

In clinical practice, wounds often present a high degree of complexity, making the selection of an appropriate dressing form crucial for effective treatment. Natural polymers can be easily manufactured into various forms, such as hydrogels, foams, hydrocolloids, and films, which are used as wound dressings. Table 4 summarizes the advantages and disadvantages of different dressing forms.

In summary, in clinical practice, personalized dressing treatment methods for patients can be comprehensively considered from the following perspectives: (1) Select dressings from appropriate sources. When dealing with natural polymer-based dressings with unstable collagen sources, it is crucial to evaluate whether the patient has allergies. (2) Select dressings with different effects, such as antibacterial, anti-inflammatory, and hemostatic properties, according to the patient’s current most urgent requirement. (3) Consider the patient’s financial situation.

## 6. Challenges and Future Prospects

Patients diagnosed with diabetes constitute the principal high-risk population for non-traumatic amputation, which significantly diminishes their quality of life [154]. Consequently, the development of novel dressings is of great significance for the progress of innovative strategies for wound treatment. This research retrospectively summarizes the sources, characteristics, clinical applications, and current advancements in diabetic wound treatment regarding collagen, gelatin, CS, HA, alginate, and cellulose. Given that collagen, gelatin, CS, HA, alginate, and cellulose all possess typical characteristics of bioactive materials, such as biocompatibility, low immunogenicity, and biodegradability, dressings based on these materials are currently a focal point of research for the treatment of diabetic skin ulcers. Meanwhile, due to space constraints, a complete review of all the literature on natural polymer-based dressings related to diabetic wounds could not be included.

Based on a retrospective and comprehensive analysis of collagen, gelatin, CS, HA, alginate, and cellulose, the following common challenges have been identified: (1) The extraction processes for these materials have not been optimized, leading to an inconsistent efficacy of bioactive materials derived from different sources. The absence of standardization poses challenges for ensuring product consistency and increases the cost of clinical translation. (2) Most studies integrating modern dressing technologies, such as tissue-engineered loadings of stem cells, exosomes, or genes, are still confined to animal experiments and have not progressed to clinical trials, thus requiring further research. (3) Most studies solely focus on therapeutic efficacy without exploring the underlying molecular mechanisms, which impedes a more in-depth exploration of causal relationships. (4) Experimental animals predominantly comprise STZ-induced diabetic mouse or rat models. However, the skin of rats and mice differs from human skin in morphology, leading to differences in the healing process [155]. This approach does not conform to the chronic characteristics of real DFUs. These experiments could additionally utilize pigs, whose skin has a closer resemblance to human skin, as animal models [156]. (5) Natural polymers are typically derived from animal and plant sources, and their extraction and purification processes vary significantly. (6) The same natural polymer dressing may exhibit poor stability due to different sources, which increases the cost of its commercialization. As a result, it is difficult to ensure consistency and stability in production for materials from different sources. Owing to the variability of biological sources, we propose implementing quality control throughout the entire production process: (1) Source control: The consistency of the source of dressings for the same product can be controlled. For example, BC can control the consistency of the microbiota. (2): Production process control: The production process for the same product should be documented comprehensively to ensure consistency in the extraction and purification process. Meanwhile, its molecular weight and mechanical structure can be tested to maintain stability. (3) Finished product control: Regulatory authorities can also conduct random inspections on products of the same type generated from different batches to ensure their biological activity and stability.

Meanwhile, it is essential to consider the influence of cost factors. For example, the main source of CS is the large amount of crustacean waste produced by food processing, which is one of the most common sources. Hence, its source cost is relatively low [157]. However, the source of collagen is generally more expensive than the source of CS, which can affect its market pricing. As is well known, patients with DFUs already have a significant medical and economic burden. Therefore, low-cost dressings will become the first choice in the treatment process. Overall, for the production of advanced natural polymers, low cost and high treatment efficiency should be regarded as important measurement indicators to promote further application in clinical practice.

As previously stated, no animal model is completely consistent with real clinical wounds currently. Therefore, wound dressings constructed based on preclinical studies may not fully adapt to changes in the wound environment. The regulatory healing mechanism of real diabetes wounds is more complex than that of animal models. Animal models cannot depict the true state of human diseases [158]. Therefore, future research should conduct in-depth investigations on diabetic wounds in a clinical setting and evaluate the possible changes during the treatment process to develop more ideal dressings.

Despite these challenges, notable progress has been achieved in recent years regarding natural polymer-based dressing for diabetic wound care. Novel technologies such as 3D printing, bioinks, and biomimetic scaffolds have emerged conspicuously. These innovations surmount critical limitations in gene, stem cell, and exosome therapies, including uneven distribution within wounds and in vitro survival time. Meanwhile, smart responsive dressings partially address the complex wound environment by regulating the release rate of loaded drugs or molecules according to wound conditions. However, the process of clinical transformation for smart hydrogel faces many challenges, including how to employ appropriate storage conditions (temperature-controlled supply chain, appropriate time, and closed container) to keep the hydrogel’s water and the drug inactive [159]. At the same time, it also faces the problem of how to preserve the activity of the hydrogel after sterilization [160]. Therefore, in the process of clinical translation, researchers need to overcome these issues in future research.

In conclusion, natural polymer-based dressings are a highly promising new type of wound dressing for diabetic wounds. Although there are difficulties in clinical translation, researchers are gradually overcoming these difficulties, accelerating clinical application, and generating positive news for the treatment of DFUs.

## Figures and Tables

**Table 2:** Registered clinical trials of natural polymers for DFUs.

The Type of Natural Polymer	Recruitment Status	NCT Number	Experimental Group	Control Group	Status of Study	Study Start (Actual)	Study Completion (Actual)	Participant (Actual/Estimated)
Collagen	Completed	NCT02427802	Gentamicin–collagen sponge	Placebo collagen sponge/no sponge group	Phase 3	May 2015	October 2016	612
		NCT00593567	Gentamicin–collagen sponge	Levofloxacin	Phase 2	December 2007	May 2009	69
		NCT05324930	Piscean collagen dressing	Saline-infused dressing	Not applicable	December 2021	September 2022	180
		NCT00235196	Collagen ORC antimicrobial matrix	Not applicable	Phase 4	July 2004	December 2005	48
		NCT01951768	Garamycin–collagen sponge	Systemic antibiotic	Phase 4	September 2013	June 2016	88
		NCT00659646	Gentamicin–collagen sponge + levofloxacin	Levofloxacin	Phase 2	April 2008	February 2010	56
		NCT05417425	Omeza collagen matrix, Omeza lidocaine lavage, Omeza skin protectant	Not applicable	Phase 1	September 2022	December 2023	25
		NCT06470087	SOC and type I collagen-based skin substitute	SOC and human amnion/chorion membrane	Not applicable	June 2024	September 2024	28
		NCT00493051	GAM501	Collagen gel/SOC	Phase 2	November 2007	December 2009	124
		NCT00065663	GAM501	Not applicable	Phase 1	August 2002	December 2004	21
		NCT07046403	High-purity type I collagen-based skin substitute and SOC	Human amnion/chorion membrane and SOC	Not applicable	July 2025	October 2025	120
	Recruiting	NCT07161830	Omeza^®^ Complete Matrix (collagen derived from whitefish skin) and SOC	SOC	Not applicable	December 2025	Not applicable	130
		NCT06618612	PuraPly AM/PuraPly XT + SOC	SOC	Not applicable	August 2024	Not applicable	170
	Terminated	NCT01108263 (sponsor terminated)	INTEGRA^TM^ flowable on wound bed	INTEGRA^TM^ flowable on wound and injected subcutaneously	Phase 4	June 2010	August 2011	5
		NCT03509870 (lack of recruitment)	Mesenchymal stromal cells in a collagen scaffold	Not applicable	Phase 1	June 2018	April 2020	2
		NCT00958711 (lack of budget)	Collagen-based, decellularized equine pericardial dressing for skin surface wounds	Gauze moistened with sterile saline	Not applicable	January 2009	May 2012	90
	Withdrawn	NCT01228500	PriMatrix + negative pressure wound therapy	PriMatrix	Not applicable	January 2008	January 2013	0
	Unknown	NCT03037970	ABSOLVE	Collagen wound dressing wetted with buffer	Phase 2	Not applicable	Not applicable	40
		NCT01537016	PROMOGRAN^®^	Tielle	Not applicable	July 2013	Not applicable	250
CS	Completed	NCT02789033	Isosorbide dinitrate spray + chitosan	Chitosan/isosorbide dinitrate spray	Phase 3	June 2015	August 2015	68
	Terminated	NCT04178525 (COVID-19 restrictions)	ChitoCare^®^ Gel	Placebo gel	Not applicable	August 2018	September 2020	46
		NCT00434538 (sponsor’s financial reasons)	BST-DermOn	SOC	Phase 3	February 2007	November 2008	40
	Unknow	NCT02413086	External herb chitosan	Traditional gauze	Not applicable	April 2015	Not applicable	320
HA	Completed	NCT06680856	HA gel + PRP gel	HA gel	Phase 2	February 2021	October 2024	72
	Recruiting	NCT07131410	Hyaluronic acid cream	10% urea cream	Not applicable	November 2024	Not applicable	83
	Unknown	NCT05198544	Hēlaquis matrix (hyaluronic acid matrix)	Not applicable	Not applicable	Not applicable	Not applicable	Not applicable
Alginate	Not yet recruiting	NCT06873646	Kelulut honey-infused alginate	Manuka honey dressing/ standard dressing product	Phase 1	Not applicable	Not applicable	110
	Completed	NCT02577900	Nanocrystalline silver alginate	Honey gel sheet/conventional dressing (Jelonet)	Not applicable	February 2013	August 2015	31
Cellulose	Terminated	NCT02667327 (sponsor terminated)	100 μM aCT1 peptide plus hydroxyethyl cellulose	Hydroxyethyl cellulose without drug/SOC	Phase 3	November 2018	May 2020	124
		NCT01849965 (not applicable)	DSC127 0.03% in hydroxyethyl cellulose (HEC) with parabens	Vehicle gel comprising HEC with parabens	Phase 3	April 2013	December 2015	396

Note: SOC: Standard of care. No registered clinical studies concerning gelatin-based dressings and BC-based dressings for diabetic wounds were identified.

**Table 3:** Traditional dressings and innovative natural polymer-based dressings.

Dressing	Advantage	Disadvantage
Traditional dressing	(1) Low cost (2) Dressings, such as cotton gauze, are non-toxic and biocompatible (3) Easy to obtain and already mass-produced	(1) Frequent dressing changes may cause secondary trauma (2) Poor absorption capacity
Novel natural polymer-based dressing	(1) Biocompatible, non-toxic, and low immunogenicity (2) Provide a moist wound environment (3) Absorbs exudate (4) Reduce secondary damage	(1) High economic burden (2) Limited availability of mature products (3) Most remain at the preclinical stage

**Table 4:** Forms of dressing [11,153].

Forms of Dressings	Advantage	Disadvantages	Applicable Clinical Scenario
Films	Transparent, easily observable, and effectively prevents bacterial contamination	Poor exudate absorption capacity and prone to infection	Dry wounds, superficial wounds
Foam	Good breathability, high plasticity, strong exudate absorption capacity	Low transparency, difficult to observe, and low biocompatibility	Deeper wounds, exudative wounds
Hydrogels	Autolytic debridement is easy to replace, has high water content, hydrates, and alleviates pain and inflammatory response	Low adhesion, requires frequent changing, and has low mechanical properties	Exudative wounds
Hydrocolloids	High density, adhesive, and excellent water absorption	Used for high-exudate wounds that cause excessive moisture and maceration	Exudative wounds

## Data Availability

No new data were created or analyzed in this study.
